# Unfolded Protein Response-Dependent Communication and Contact among Endoplasmic Reticulum, Mitochondria, and Plasma Membrane

**DOI:** 10.3390/ijms19103215

**Published:** 2018-10-18

**Authors:** Atsushi Saito, Kazunori Imaizumi

**Affiliations:** 1 Department of Stress Protein Processing, Institute of Biomedical & Health Sciences, Hiroshima University, 1-2-3 Kasumi, Minami-ku, Hiroshima 734-8553, Japan; 2 Department of Biochemistry, Institute of Biomedical & Health Sciences, Hiroshima University, 1-2-3 Kasumi, Minami-ku, Hiroshima 734-8553, Japan; imaizumi@hiroshima-u.ac.jp

**Keywords:** unfolded protein response, ER morphology, mitochondria-associated ER membrane, ER-PM contact sites

## Abstract

The function of the endoplasmic reticulum (ER) can be impaired by changes to the extra- and intracellular environment, such as disruption of calcium homeostasis, expression of mutated proteins, and oxidative stress. In response to disruptions to ER homeostasis, eukaryotic cells activate canonical branches of signal transduction cascades, collectively termed the unfolded protein response (UPR). The UPR functions to remove or recover the activity of misfolded proteins that accumulated in the ER and to avoid irreversible cellular damage. Additionally, the UPR plays unique physiological roles in the regulation of diverse cellular events, including cell differentiation and development and lipid biosynthesis. Recent studies have shown that these important cellular events are also regulated by contact and communication among organelles. These reports suggest strong involvement among the UPR, organelle communication, and regulation of cellular homeostasis. However, the precise mechanisms for the formation of contact sites and the regulation of ER dynamics by the UPR remain unresolved. In this review, we summarize the current understanding of how the UPR regulates morphological changes to the ER and the formation of contact sites between the ER and other organelles. We also review how UPR-dependent connections between the ER and other organelles affect cellular and physiological functions.

## 1. Introduction

The endoplasmic reticulum (ER) is the intracellular organelle responsible for the synthesis, folding, modification, and assembly of secretory proteins. This organelle has a unique system that maintains an optimal environment for protein quality control and is collectively known as the unfolded protein response (UPR) [1,2]. Accumulation of unfolded and/or misfolded proteins in the ER lumen leads to ER dysfunction and apoptotic cell death [3]. The UPR is activated to resolve protein misfolding events and thus ameliorate the ER environment and maintain homeostasis [4]. Additionally, the function of the UPR has been extended from maintenance of protein quality control to include fine-tuning of cellular homeostasis and biological functions, such as cell development, differentiation, glycogenesis, and lipid metabolism [5,6,7,8,9,10]. Previous reports have indicated that regulation of these physiological processes is controlled by signal transduction events derived from the ER, including the UPR [11,12,13,14,15]. Moreover, morphological changes and dynamics of the developed ER also manipulate cellular homeostasis through communication with other organelles [16,17,18,19]. Here, we discuss current knowledge of the diverse functions and prospects of the UPR for regulating ER morphology and the formation of contact sites with mitochondria and the plasma membrane (PM).

## 2. ER Stress Transducers and Key Molecules of the UPR

The ER is a critical organelle for lipid synthesis, calcium storage, protein synthesis, and post-translational modifications of many secretory and membrane proteins. An altered environment in the ER and/or cellular malfunction, such as calcium depletion in the ER lumen, expression of mutated proteins, oxidative stress, and ischemia, cause accumulation of unfolded and/or misfolded proteins in the ER lumen, which perturbs ER functions. These abnormal conditions are collectively known as ER stress [1,2,3]. Three major canonical ER stress transducers, inositol-requiring kinase 1 (IRE1) [20], protein kinase R-like ER kinase (PERK) [21], and activating transcription factor 6 (ATF6) [22], are activated in response to ER stress. These three proteins initiate signaling events that induce the expression of chaperone molecules, attenuate protein translation, and degrade unfolded proteins, and collectively these are referred to as the UPR [1,2,4]. These ER stress transducers localize at the ER membrane. ER stress triggers IRE1 dimerization and trans-autophosphorylation [20]. Activated IRE1 processes a 26-nucleotide intron of the x-box binding protein 1 (Xbp1u) mRNA (unspliced form of XBP1) via its RNase activity. The splicing produces the mature XBP1s mRNA (spliced form of XBP1) [23,24,25], generating the transcription factor, XBP1s. The target genes of XBP1s include molecular chaperones and ER-associated degradation (ERAD)-related genes [25]. PERK also oligomerizes and auto-phosphorylates in response to ER stress. Phosphorylated PERK directly phosphorylates the α subunit of eukaryotic initiation factor 2 (eIF2α). The phosphorylated eIF2α accelerates the disassembly of the 80S ribosome, and eventually this process suppresses global protein synthesis [21,26,27]. Anomalistically, ATF4 escapes translational attenuation by phosphorylated eIF2α. The gene coding for ATF4 has open reading frames (ORFs) in its 5′-untranslated region. These upstream ORFs prevent translation of native ATF4 under normal conditions. The phosphorylation of eIF2α and the disassembly of the 80S ribosome circumvent these pseudo ORFs and promote the translation of native ATF4 [26,28]. ATF4 drives the expression of several genes involved in amino acid metabolism [26,29,30]. In turn, ATF4 induces the transcription of the transcription factor, CCAAT enhancer binding protein homologous protein (CHOP) [31,32,33]. Prolonged expression of CHOP induces cell death [34]. ATF6 translocates from the ER to the Golgi apparatus following ER stress. This protein undergoes subsequent processing by site-1 and site-2 proteases [35,36], and the cleaved N-terminal fragments move into the nucleus. The ATF6 N-terminal fragments act as transcription factors and induce the expression of ER molecular chaperones, such as binding immunoglobulin protein (BiP) [22,37]. Interestingly, recent studies have revealed that the UPR can also be activated in the absence of ER stress and prior to the accumulation of misfolded proteins. The activation of XBP1 in B cells is a differentiation-dependent event [6]. The stabilization of aggregated proteins by cross-linking using bis[sulfosuccinimidyl]suberate (BS^3^) did not show aggregated proteins in hen egg lysozyme (HEL)-specific B cell receptor (BCR) transgenic (MD4) cells [38,39]. The data suggest that the activation of XBP1 during the differentiation of B cells to plasma cells may occur in a stress- and misfolded protein-independent manner, and be initiated by signaling of the BCR to control the differentiation program. These phenomena are also observed in macrophages. Optimal secretion of pro-inflammatory cytokines by the Toll-like receptor (TLR) is mediated by XBP1, in which, TLR stimulation leads to the activation of IRE1 through NADPH oxidase 2 signaling without the induction of global ER stress markers [40]. Glucose-stimulated activation of IRE1 regulates glucose levels in the absence of ER stress in pancreatic β-cells [41,42]. Vascular endothelial growth factor (VEGF) activates all UPR pathways via phospholipase C type γ (PLCγ)-mediated crosstalk with the mammalian target of rapamycin (mTOR) complex 1 (mTORC1) in an ER stress-independent manner, contributing to the survival and angiogenesis of endothelial cells [43]. Therefore, ER stress- and misfolded protein-independent activation of the UPR is closely involved in the regulation of biological functions and cellular homeostasis [5,44]. These beneficial outcomes are also regulated by morphological changes to the ER and communication between the ER network and other organelles [16,17,18]. Thus, the UPR may orchestrate overall cellular homeostasis through ER dynamics and cooperative attachment among organelles. Perturbation of these systems can lead to the pathogenesis of various diseases, including neurodegenerative diseases.

## 3. Morphological Changes to the ER by the UPR

The regulation of ER biogenesis and expansion is controlled by the UPR. ER expansion is necessary to increase the protein folding capacity, which handles unfolded proteins that accumulate in the ER lumen [45,46]. XBP1, the downstream transcription factor of IRE1, is mainly responsible for ensuring sufficient regulation of ER biogenesis, including an increase in the biosynthesis of ER proteins and lipid biogenesis [47,48]. Transformation of B cells to plasma cells accelerates membrane biogenesis and exponential expansion of the ER, which allows these cells to secrete large quantities of immunoglobulins [49,50]. The selective deletion of *Xbp1* in B cells does not cause a change in the levels of phosphatidylethanolamine (PtdEtn), phosphatidylserine, and phosphatidylglycerol when compared with those found in wild-type B cells [51]. In contrast, significant decreases in the levels of phosphatidylcholine (PtdCho), sphingomyelin (SM), and phosphatidylinositol are observed in these *Xbp1*-deficient cells. In addition, ER expansion following lipopolysaccharide (LPS)-stimulated activation is inhibited in *Xbp1*-deficient B cells [47]. Thus, XBP1 drives morphological changes to the ER by regulating the amounts of membrane components, including PtdCho. The morphological changes to the ER are most drastically affected by the biosynthesis of PtdCho, because PtdCho is the most abundant cellular phospholipid and a major component of ER membranes [52]. PtdCho is primarily produced from cytidine diphosphocholine (CDP-choline) [52]. Choline cytidylyltransferase (CCT) converts phosphocholine to CDP-choline in the presence of cytidine triphosphate (CTP) [53]. The residual phosphocholine is transferred to diacylglycerol (DAG), yielding PtdCho [52]. Cholinephosphotransferase (CPT1) [54] or choline/ethanolaminephosphotransferase (CEPT1) [55] catalyze this final step. The level and synthesis of CCT is upregulated in fibroblasts overexpressing a spliced form of XBP1 [56]. The increase in activity of CCT accelerates the production of PtdCho. Increasing the synthesis of PtdCho by transduction of CCT is only sufficient for a minor expansion of the rough ER. In contrast, the transduction of the spliced form of XBP1 yields a clear increase in PtdCho synthesis and promotes expression of abundant ER proteins, which leads to robust expansion of the ER. Therefore, XBP1 may orchestrate ER biogenesis by coordinating phospholipid biosynthesis and the expression of ER proteins. ATF6 also plays roles in driving ER biogenesis and its expansion. The expression of a green fluorescence protein (GFP) fusion construct anchored to the ER membrane by the C-terminal tail of cytochrome *b*(5) (GFP-b(5)tail) induces expansion of the ER [57]. Only the ATF6 branch is activated in response to the expression of the GFP-b(5)tail, without upregulation of known target genes of ATF6 [58]. GFP-b(5)tail-dependent activation of ATF6 may be regulated by a sensing mechanism within the lipid bilayer, but not the ER lumen because the GFP-b(5)tail lacks a luminal domain. Overexpression of the N-terminal fragments of ATF6 induces PtdCho biogenesis and modulates the CDP-choline pathway, leading to the expansion of the ER [59]. This ATF6-induced ER expansion can occur in the absence of XBP1, indicating that the ATF6 pathway regulates lipid production and ER biogenesis independent of the XBP1 pathway. These ER expansions and morphological changes by UPR components may facilitate communication and contact between the ER and other organelles.

## 4. Mitochondria-Associated ER Membrane (MAM) and UPR

The ER is physically and biologically connected to mitochondria. A specialized subdomain of the ER is called the mitochondria-associated ER membrane (MAM) [19]. MAM is an intracellular lipid raft-like structure intimately involved in calcium homeostasis, lipid metabolism, apoptosis, and mitochondrial functions [19,60,61]. In mammalian cells, several types of connectors for MAM have been identified. Mitofusin 2 (MFN2) is a dynamin-related GTPase localized at the ER surface and mitochondria [62]. MFN2 contributes to tethering between the ER and mitochondria by the homologous interaction of ER-associated MFN2 with mitochondrial MFN2. MFN2 also forms a heterologous interaction with MFN1, a homologue protein only localized at the outer mitochondrial membrane [62]. Vesicle-associated membrane protein (VAMP)-associated protein B (VAPB), an ER protein, also forms a connection between the ER and mitochondria by interacting with protein tyrosine phosphatase-interacting protein 51 (PTPIP51), which is localized at mitochondria [63]. ER-resident 75 kDa glucose-regulated protein (GRP75) and mitochondrial voltage-dependent anion channel 1 (VDAC1) form a complex with subtype 3 of the 1, 4, 5-triphosphate receptor (IP3R3). This complex serves as a calcium exchange platform at MAM [64]. B-cell receptor-associated protein 31 (BAP31), localized at the ER, interacts with mitochondrial fission 1 homolog (FIS1) and phosphofurin acidic cluster sorting protein-2 (PACS-2) as MAM connectors related to the induction of apoptosis [65,66]. Multiple molecules involved in protein quality control, autophagy, mitochondrial dynamics, lipid synthesis, and the inflammasome, are recruited to MAM, suggesting that diverse signaling derived from MAM orchestrates these physiological events [67,68,69,70].

Dysfunctions of MAM may be responsible for the pathogenesis of several diseases, including metabolic and neurodegenerative diseases [71,72]. Glucose homeostasis is tightly regulated by the liver, and both metabolic inflexibility and insulin resistance lead to the development of hepatic metabolic diseases. The functions of the developed ER and mitochondria, which play a central role in the regulation of hepatic metabolism, and their contact sites are also related to those events in the liver. Many recent studies have suggested that MAM is an important convergence point for regulating hormonal and nutrient signaling in the liver [73,74]. In addition, several proteins in the insulin signaling pathway, such as Akt and mTORC2, are located in MAM and manipulate insulin signaling [75]. ER-mitochondria miscommunication leads to hepatic insulin resistance, which may trigger the pathogenesis of type 2 diabetes mellitus [76]. Dysfunction of MAM is also involved in the development of neurodegenerative diseases, including Parkinson’s disease (PD) [77]. PD-related genes have been identified as regulators of mitochondrial function, and PD-associated mutations in these genes lead to mitochondrial dysfunction. The α-Synuclein (SNCA) is linked to familial and sporadic PD. SNCA is enriched in the MAM fraction of the ER in mouse and human brain tissue [78]. Two other PD-related proteins, Parkin and phosphatase and tensin homolog (PTEN)-induced kinase 1 (PINK1), are also involved in mitochondrial regulation. These proteins control mitochondrial degradation by mitophagy, a selective autophagy that eliminates old and damaged mitochondria [79]. MAM is known to constitute initiation sites for this process [80]. Parkin is enriched in the MAM fraction of neurons exposed to glutamate excitotoxicity [81]. PINK1 is also found in the MAM fraction during mitophagy [82]. Although detailed involvement between MAM functions and PD remains unclear, future work should aim to define how the dysfunction of MAM promotes the pathogenesis of PD.

The induction of various MAM proteins correlates with ER stress, and of those proteins, Rab32 is a GTPase that localizes to the ER and mitochondria [83,84]. Rab32 regulates ER-mitochondria interactions and mitochondrial dynamics [85]. The expression of Rab32 is upregulated upon brain inflammation in a mouse model and lesions of multiple sclerosis (MS) brain tissues [86,87]. The treatment of human neuroblastoma SH-SY5Y cells with ER stress inducers leads to transcriptional activation of Rab32, indicating that the expression of Rab32 may be under the control of the ER stress-induced UPR. The induction of Rab32 in vivo parallels those of ER stress-related genes in active lesions of the MS brain [87]. Rab32 is also known to modulate MAM properties [84]. The expression of the other MAM regulatory proteins, including MFN2, GRP75, and PACS-2, are upregulated in active lesions of the MS brain, which is consistent with the upregulation of Rab32 expression [87]. Overexpression of Rab32 or expression of the dominant-active form of Rab32 in SH-SY5Y cells causes impaired mitochondrial dynamics and distribution and the inhibition of neurite outgrowth. UPR-dependent Rab32 may manipulate neurite outgrowth via regulation of mitochondrial dynamics and the formation of MAM.

Sigma 1 receptor (Sig1R) is another MAM protein that is a chaperone protein highly expressed in spinal motor neurons and several peripheral organs, including the lung, kidney, liver, pancreas, spleen, adrenal gland, and heart [88,89,90]. Sig1R specifically localizes at MAM and forms a complex with ER chaperones under normal conditions. Calcium depletion in the ER lumen accelerates the dissociation of Sig1R from ER chaperones, followed by regulation of a variety of cellular functions, such as the transfer of calcium signaling between the ER and mitochondria [89,91]. A recent study has shown that *Sig1r*-deficiency attenuates ER-mitochondria crosstalk and triggers the degradation of moderate motor neurons in *Sig1r*-deficient mice, suggesting that Sig1R is a key factor for ensuring the integrity of MAM [92]. *Sig1r*-deficiency disrupts ER-mitochondria contacts, which impairs intracellular calcium signaling and mitochondrial dynamics and transport. Interestingly, the expression of Sig1R is upregulated in response to ER stress [93]. Sig1R is transcriptionally upregulated by treatment with ER stress inducers. Many transcription factors, including XBP1 (IRE1 pathway), ATF4 (PERK pathway), and ATF6 (ATF6 pathway), are activated downstream of the UPR. Knockdown experiments indicate that suppressing the expression of ATF4 decreases the level of Sig1R. Furthermore, strong binding of ATF4 to a 5′ upstream region of *Sig1r* was observed by a chromatin immunoprecipitation assay, suggesting that the expression of Sig1R is regulated by the PERK pathway of a UPR branch. These previous observations indicate that the UPR may fine-tune the functions of MAM through PERK pathway-dependent expression of Sig1R, whereas Sig1R can regulate UPR through direct interaction with ER stress transducers. Sig1R binds to the monomeric form of IRE1 at MAM under ER stress conditions [94]. The interaction of Sig1R with IRE1 leads to IRE1 adopting an active-state conformation. Although these events transiently interfere with dimerization and autophosphorylation of IRE1, the stabilized active form of IRE1 is able to exert long-lasting activation. Knockdown of *Sig1r* disrupts IRE1-XBP1 signaling, resulting in the induction of apoptosis by ER stress. The report suggests that the stabilization of IRE1 by Sig1R at MAM serves as a resistance against ER stress by ensuring long-lasting activation of IRE1-XBP1 signaling.

Recent studies have reported that Sig1R may be involved in the etiology of neurodegenerative diseases. Alzheimer’s disease (AD) is now accepted as being caused by amyloid β (Aβ) plaques and tau neurofibrillary tangles [95,96]. Aβ is generated at MAM and may affect the functions of the ER, mitochondria, and MAM [97]. Knockdown of *Sig1r* in hippocampal neurons causes neuronal degeneration. The uncontrolled expression of Sig1R leads to abnormal calcium shuttling from the ER to mitochondria [97]. Additionally, impaired expression of Sig1R is observed in the brain of APP_Swe/Lon_ mice, the AD mouse model (Swedish (K670/M671) and London (V717I) mutations) [98], and postmortem cortical brain tissue of AD patients. Downregulation of Sig1R is also detected in putamen of PD and in the lumbar spinal cord of amyotrophic lateral sclerosis (ALS) patients [99,100]. *Sig1r*-knockdown cells exhibit vulnerability to dopamine toxicity, which is involved in the etiology of PD, resulting in the induction of apoptosis [101]. *Sig1r*-deficient mice show muscle weakness and loss of motor neurons [92]. The pathogenesis is similar to those of ALS. *Sig1r*-deficiency triggers impaired mitochondrial fission and transport in axons, leading to axonal degeneration. The pathogenesis of these neurodegenerative diseases involves the induction of ER stress and the UPR [102]. Thus, disturbance of the UPR-Sig1R axis may cause aberrant functions of MAM, resulting in the development of these diseases.

**Figure 1:**
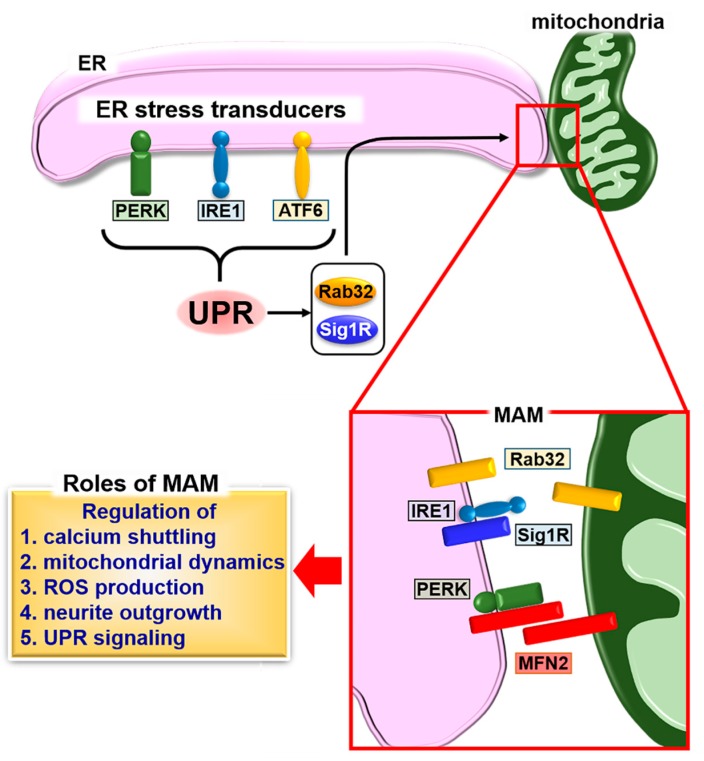
Schematic describing the formation of mitochondria-associated endoplasmic reticulum (ER) membrane (MAM) and the unfolded protein response (UPR). The UPR induces the expression of MAM connectors, Rab32 and sigma 1 receptor (Sig1R), followed by fine-tuning of calcium signaling, calcium shuttling, mitochondrial dynamics, reactive oxygen species (ROS) production, and neurite outgrowth through the formation of MAM. The stability of inositol-requiring kinase 1 (IRE1) is regulated by Sig1R binding. The binding of Sig1R to IRE1 leads to long-lasting activation of IRE1, which promotes cellular survival under ER stress conditions. A second MAM connector, mitofusin 2 (MFN2), interacts with protein kinase R-like ER kinase (PERK) to inhibit its activity for regulating ROS production, calcium shuttling, and mitochondrial morphology.

In contrast to the regulation of MAM by UPR-related molecules, several MAM connectors can modulate the UPR. The three branches (IRE1, PERK, and ATF6 pathways) of the UPR that are induced by ER stress show excessive activation in *Mfn2*-deficient mouse embryonic fibroblasts [103]. The over-activation of IRE1 and PERK pathways triggers the attenuation of ER stress-dependent apoptosis and autophagy, respectively, in these deficient cells. MFN2 interacts with PERK to suppress its activation under normal conditions. Loss of function of MFN2 causes an increase in reactive oxygen species (ROS) production, mitochondrial calcium overload, and impaired mitochondrial morphology through the sustained activation of PERK. These data suggest that the MAM connector, MFN2, has unique roles in cooperatively orchestrating mitochondrial dynamics and the UPR. Additionally, PERK localized at MAM is also known as a MAM connector [104]. This connector promotes apoptosis following insults, requiring the transfer of ROS-mediated signals between the ER and mitochondria. Accelerated ROS production and the over-activation of PERK because of *Mfn2* deficiency, and an increase in ROS damage by PERK localized at MAM may synergistically accelerate ROS-based apoptosis. Consequently, the UPR and MAM may have bidirectional communication that enables regulation of the ER and mitochondrial dynamics and cellular homeostasis (Figure 1).

## 5. ER-PM Contact Sites and the UPR

Regions of the ER closely to the PM (the distance is typically within 10–30 nm) were first revealed by electron microscopy data [105]. ER-PM contact sites have emerged as key regulators of intracellular calcium dynamics [106,107,108,109]. Previous studies have shown that calcium depletion in the ER lumen triggers extracellular calcium influx through the PM at ER-PM contact sites to replenish the calcium concentration of the ER lumen [110,111]. Stromal-interacting molecule 1 (STIM1) is an integral ER protein that regulates the formation of ER-PM contact sites in response to calcium depletion in the luminal ER [112]. STIM1 senses a decrease in the intraluminal ER calcium level and undergoes conformational changes. The exposed domains following the conformational change to STIM1 preferentially target phosphatidylinositol 4,5-biphosphate (PI(4,5)P2) enriched ER-PM contact sites [110,113,114]. STIM1 directly recruits and forms a complex with Orai1, calcium channels localized at the PM, followed by replenishing calcium levels in the ER lumen [110,115].

Tethering molecules are necessary to form ER-PM contact sites. Previous reports have identified at least four protein families as ER-PM tethering proteins in yeast and/or metazoans: Extended-synaptotagmins (E-Syts) (Tricalbins in yeast), VAPs (Scs2/22 in yeast), transmembrane protein 16 (Tmem16) (Ist2 in yeast), and junctophilins (JPHs) [116,117,118,119]. Each of these protein families have been independently studied for their own cellular pathways, suggesting that ER-PM contact sites function as hubs for numerous cellular signal transduction events. Recent studies have indicated that dysfunction of tethering events may disturb cellular functions. JPHs were identified by screening using monoclonal antibodies generated from mice immunized with membrane vesicles of rabbit skeletal muscles [119]. There are four junctophilins in mammals. These four JPHs have been studied mainly in the context of cell physiology in skeletal muscle. Four JPHs are selectively expressed in several tissues. JPH1 and JPH2 are preferentially expressed in heart and skeletal muscle, whereas JPH3 and JPH4 are highly expressed in brain and neuronal tissues [119,120]. The C-terminal transmembrane domains of JPHs are anchored to the sarcoplasmic reticulum (SR) and the N-termini of JPHs contain basic membrane occupation and recognition nexus (MORN) domains. The MORN domain binds to phospholipids, including phosphatidylserine and phosphatidylinositol (3,4,5)-trisphosphate [121]. In addition to those phospholipids, a previous report suggested that the purified junctophilins bind to electrostatically neutral lipids, such as PtdCho and SM [121]. Malfunctioning ER-PM contact sites formed by JPHs impairs calcium transients in cardiac myocytes. These abnormalities raise the possibility of weak heartbeats, cardiac arrest, and eventual embryonic lethality in *Jph2*-deficient mice [119]. In addition, myotubes lacking JPH1 also exhibit severe reduction in store-operated calcium entry (SOCE) occurring at ER-PM contact sites, low basal cytosolic calcium levels, and low SR calcium storage [122,123]. These studies indicate that the formation of ER-PM contact sites and SOCE mediated by ER-PM contact sites are important for regulating calcium signaling in muscle cells.

The importance of ER-PM contact sites and SOCE has also been demonstrated in many other genetic studies. Heterozygous mice with the STIM1-null mutation normally grow to adulthood. In contrast, a majority (approximately 70%) of mice lacking STIM1 die within a few hours after birth [124]. These mice show cyanosis before death caused by a cardiopulmonary defect. Surviving *Stim1*-deficient mice display significant growth retardation. The weight of the deficient mice achieves approximately half that of wild-type littermates at three and seven weeks of age. Moreover, mice lacking functional STIM1 exhibit muscle fatigue and skeletal myopathy [125]. Mice without Orai1 display phenotypes similar to those observed for *Stim1*-deficient mice [126]. In addition to muscle cell dysfunction, these deficient mice (*Stim1*- and *Orai1*-deficient mice) lack immune cell functions, because activation of immune cells is closely linked with SOCE. Mast cells lacking STIM1 exhibit impaired activation of nuclear factor of activated T-cells (NFAT), which plays central roles in transcriptional regulation of cytokines [127], suppressed degranulation, and in reducing cytokine production [128]. These abnormalities in *Stim1*-deficient mast cells are also observed in *Orai1*-deficient mast cells [129]. The reduction of SOCE and inhibition of cytokine production are found not only in mast cells from *Stim1*- and *Orai1*-deficient mice, but also in T cells [130,131]. Selective ablation of STIM1 in T cells shows a lymphoproliferative phenotype and a selective decrease in regulatory T cells [130]. These phenotypes in immune cells of *Stim1*- and *Orai1*-deficient mice are consistent with those of the severe combined immunodeficiency (SCID) symptoms found in human patients who have mutations in the *Stim1* or *Orai1* gene, resulting in abnormal STIM1 or Orai1 functions [126]. These genetic studies and clinical observations indicate the importance of the formation of ER-PM contact sites and SOCE via the contact sites in the activation and maintenance of immune cells, as well as functional regulation of muscle cells.

Morphological changes to the ER and regulation of ER dynamics are essential for the efficient formation of ER-PM contact sites. ER dynamics are regulated by microtubule- and actin-binding proteins [132,133]. Of those proteins, filamin A (FLNA) is known as a connector between the ER and actin cytoskeleton [132]. PERK has been identified as an interacting partner of FLNA at the ER membrane by a proximity-dependent biotin identification (BioID) assay [134]. PERK acts as a scaffold molecule for FLNA, enabling interlocking between F-actin and ER dynamics. The dimerization of PERK induced by calcium depletion in the ER lumen is necessary for the interaction between PERK and FLNA. Simultaneously, the decrease in calcium concentration in the ER lumen leads to activation of STIM1. The PERK-FLNA axis accelerates remodeling and alters the polymerization dynamics of F-actin, followed by the relocalization of cortical ER containing STIM1 to the PM. These morphological changes to the ER are regulated by the PERK-FLNA connection and allow for the efficient formation of ER-PM contact sites and calcium influx to replenish the luminal calcium level. Thus, PERK manipulates morphological changes to the ER and the formation of ER-PM contact sites by its dimerization, but not via signal transduction.

**Figure 2:**
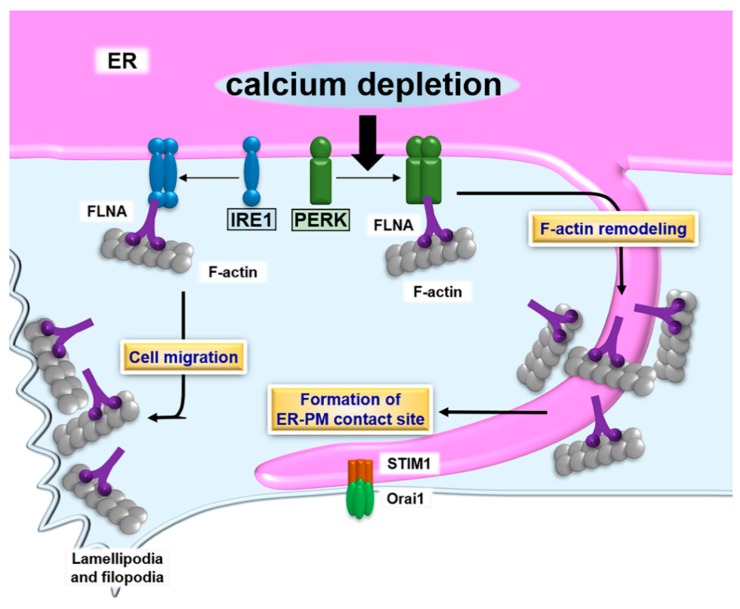
Schematic describing the formation of ER- plasma membrane (PM) contact sites and the UPR. PERK dimerizes in response to calcium depletion in the ER lumen. The PERK dimer binds to filamin A (FLNA), followed by accelerating F-actin remodeling and formation of ER-PM contact sites containing stromal-interacting molecule 1 (STIM1) and Orai1. The contact sites promote calcium influx, which restores the calcium level to a normal value in the ER lumen. The IRE1 dimer also interacts with FLNA. Although the effect of the IRE1-FLNA interaction on the formation of ER-PM contact sites remains unclear, the binding of IRE1 to FLNA does modulate ER dynamics and cell migration.

The other ER stress transducer, IRE1, is also involved in cytoskeleton remodeling via interaction with FLNA. A yeast two-hybrid screen identified that FLNA binds to the cytosolic domain of IRE1 [135]. Dimerization of IRE1 is an essential step for physiological interaction with FLNA. The IRE1 dimer acts as a scaffold molecule, and recruits FLNA and protein kinase C type α (PKCα). FLNA is phosphorylated by PKCα, followed by an increase in the remodeling of the actin cytoskeleton, and cell migration. The interaction between IRE1 and FLNA implicates significant roles for ER functions and ER-derived signaling, including the UPR at lamellipodia and filopodia, where active actin dynamics are observed. Additionally, the connection between IRE1 and FLNA implies the involvement of IRE1 in the formation of ER-PM contact sites, like those of PERK. As mentioned in Section 3, the IRE1 pathway is responsible for the regulation of ER biogenesis through manipulation of lipid biosynthesis. ER-PM contact sites play roles in the supply of membrane lipids from the ER membrane to the PM [106]. Lipid transfer at ER-PM contact sites may facilitate an extension of the PM and alter cellular morphology. The IRE1 pathway may bidirectionally regulate the formation of ER-PM contact sites via modulation of actin dynamics and the supply of membrane lipids at contact sites via fine-tuning of lipid biosynthesis. Collectively, these reports on PERK and IRE1 serve as reminders to the importance of defining the interactome of ER stress transducers (Figure 2).

Recent studies have indicated that morphological changes to the ER occur during cellular senescence [136]. Senescent normal human dermal fibroblasts (NHDFs) exhibit ER expansion and activation of the UPR [136]. Knockdown of *Atf6* inhibits ER expansion and the modification of senescence-associated cell shape, and decreases senescence-associated β-galactosidase activity. The ATF6-induced senescence in NHDFs may be mediated by signals through the activation of the cyclooxygenase 2 (COX2)-prostaglandin E_2_ (PGE_2_)-prostaglandin E receptor 3 (EP3) intracrine pathway [137]. Conversely, ATF6-dependent ER expansion and modification of cell shapes during senescence may be linked to remodeling of the intermediate filament vimentin [136]. The increase in expression of vimentin leads to the formation of longer and regulatory packed bundles in senescent fibroblasts [138,139,140]. Silencing *Atf6* yields lower expression of vimentin [141], suggesting that the remodeling of vimentin is, in part, under the control of ATF6. These reports implicate the possibility that ATF6 may contribute to ER expansion and modification of cell shapes via regulation of vimentin remodeling. Taking an overarching view, current understanding of the links among the UPR, formation of ER-PM contact sites, and regulation of the cytoskeleton is still developing. Further studies will likely uncover the unexpected mechanisms for cooperative regulation among UPR, ER-PM contact sites, and cytoskeletal dynamics.

## 6. Concluding Remarks

Understanding how ER morphology is regulated is essential for understanding how the ER communicates with other organelles. Moreover, characterizing this regulation is important for comprehending cellular homeostasis because a well-developed ER that spans the cytoplasm frequently changes its morphology and plays roles in bidirectional signal transmission through the formation of contact sites. These organelle contacts regulate the dynamics of each organelle and signal transduction, and ultimately orchestrate cellular homeostasis and biological functions. The UPR is a major signaling system found in the ER and is involved in regulating morphological changes to the ER. The mechanisms that regulate contact sites between the ER and other organelles are not fully understood. However, further studies will uncover new signaling cascades and concepts for organelle communication that is regulated by the UPR. Although not discussed in this review, research has also focused on characterizing contact sites between the ER and cellular components other than mitochondria and the PM (e.g., endosome and Golgi apparatus). Such studies may provide novel information that further elucidates the comprehensive regulation of cellular functions by the ER and UPR. These types of approaches may provide insight into new therapeutic targets for a variety of diseases, including neurodegenerative diseases associated with the UPR and the organelle contact sites. The expansion of a broad spectrum of UPR signaling to the regulation of morphological changes and contact sites should assist with deciphering the mechanisms for manipulating an intricate organelle network, which may lead to breakthroughs in therapeutic strategies that target various diseases.
